# Associations between ethnicity, social contact, and pneumococcal carriage three years post-PCV10 in Fiji

**DOI:** 10.1016/j.vaccine.2019.10.030

**Published:** 2020-01-10

**Authors:** Eleanor F.G. Neal, Stefan Flasche, Cattram D. Nguyen, F. Tupou Ratu, Eileen M. Dunne, Lanieta Koyamaibole, Rita Reyburn, Eric Rafai, Mike Kama, Belinda D. Ortika, Laura K. Boelsen, Joseph Kado, Lisi Tikoduadua, Rachel Devi, Evelyn Tuivaga, Catherine Satzke, E. Kim Mulholland, W. John Edmunds, Fiona M. Russell

**Affiliations:** a Infection and Immunity, Murdoch Children’s Research Institute, Parkville, Victoria, Australia; b Centre for International Child Health, Department of Paediatrics, The University of Melbourne, Parkville, Victoria, Australia; c Department of Infectious Disease Epidemiology, London School of Hygiene and Tropical Medicine, London, United Kingdom; d Department of Paediatrics, The University of Melbourne, Parkville, Victoria, Australia; e Ministry of Health and Medical Services, Suva, Fiji; f College of Medicine Nursing and Health Sciences, Fiji National University, Suva, Fiji; g Telethon Kids Institute, University of Western Australia, Nedlands, Western Australia, Australia; h Department of Microbiology and Immunology, The University of Melbourne at the Peter Doherty Institute for Infection and Immunity, Parkville, Victoria, Australia

**Keywords:** Social contact, Pneumococcal, Carriage, PCV10, Indigenous, Density, CI, confidence interval, FID, Fijians of Indian Descent, GEE, generalized estimating equations, GE/ml, genome equivalents per mil, IQR, inter-quartile range, LMICs, low- and middle-income countries, non-PCV10, non-10 valent pneumococcal conjugate vaccine, PCV, pneumococcal conjugate vaccine, PCV10, 10-valent pneumococcal conjugate vaccine, qPCR, quantitative polymerase chain reaction, URTI, upper respiratory tract infection, WHO, World Health Organization

## Abstract

**Background:**

Pneumococcal carriage is a prerequisite for pneumococcal disease. Little is known about whether social contact frequency and intensity are associated with pneumococcal carriage. In Fiji, indigenous iTaukei have higher prevalence of pneumococcal carriage compared with Fijians of Indian Descent (FID). We hypothesised that contact differences may contribute to ethnic differences in pneumococcal carriage prevalence and density.

**Methods:**

In 2015, young infants (5–8 weeks), toddlers (12–23 months), children (2–6 years), and caregivers from Suva and surrounding areas, participated in a cross-sectional survey (n = 2014), three years post pneumococcal conjugate vaccine introduction. Demographic and contact data, and nasopharyngeal swabs were collected. Pneumococci were detected, and quantified using quantitative real-time PCR, with molecular serotyping by microarray. Associations between ethnicity, contact, and pneumococcal carriage and density were estimated using multivariable generalised estimating equation regression models.

**Results:**

iTaukei participants had larger household sizes, higher pneumococcal carriage rates, more contacts, and more frequent contacts of longer duration, compared with FID. The odds of vaccine-type carriage increased by 28% (95% CI 8–53%) *P* < 0.01 in association with physical contact with 7–14 year old children. iTaukei ethnicity was associated with vaccine-type carriage (aOR) 1.73; 95% CI 1.06–2.82, *P* = 0.03) and non-vaccine type carriage (aOR 5.98; 95% CI 4.47–8.00, *P* < 0.01). Ethnicity and contact were not associated with pneumococcal density.

**Conclusions:**

iTaukei had greater frequency and intensity of contact compared with FID. Physical contact was associated with pneumococcal carriage. Observed differences in pneumococcal nasopharyngeal carriage prevalence between iTaukei and FID were not explained by differences in social contact patterns by ethnicity.

## Highlights

• Pneumococcal carriage rates and the frequency of physical contact differed by ethnicity.
• Contact with older children and toddlers was positively associated with vaccine-type carriage.
• Unvaccinated older children may become a vaccine-type carriage reservoir post-PCV10.
• Ethnic differences in social contact did not explain ethnic differences in carriage.
• Pneumococcal density was not associated with ethnicity, contact, or PCV10 status.

## 1 Introduction

*Streptococcus pneumoniae* is a leading cause of morbidity and mortality in children under five years [1]. Most pneumococcal carriers are asymptomatic, yet carriage is a prerequisite for pneumococcal disease [2]. Dense pneumococcal carriage is associated with severity of pneumococcal pneumonia [2], [3], [4], [5]. Toddlers have been considered the main pneumococcal transmission reservoir [6]. Transmission models have suggested that both toddlers and older children are involved in pneumococcal transmission [7], [8]. Inclusion of pneumococcal conjugate vaccines (PCV) in infant immunization schedules reduces carriage and transmission of vaccine-type pneumococci, and is a cost-effective means of providing direct and indirect protection against pneumococcal disease [9], [10], [11].

In 2015, there were over nine million cases of pneumococcal disease in children under five years old, and more than 300,000 of these cases died [1]. The burden of pneumococcal disease differs by age and region [1].The majority of cases occur in low- and middle-income countries (LMICs) [12]. Moreover, substantial differences exist in the incidence of pneumococcal disease, and the impact of PCV between ethnic groups within the same country, particularly between indigenous and non-indigenous populations [13], [14].

Numerous respiratory pathogens spread through close social contact, via the transmission of respiratory droplets [15], [16]. While it has been assumed that contact, either physical or non-physical, drives pneumococcal transmission, there is little empirical evidence. One study from rural South West Uganda found that the highest rates of contact tended to be between people of similar ages (age-assortative), that contact from and with children younger than 10 years involved more close contact (compared with contacts between people 10 years and older), and that the frequency of close contacts increased the age-adjusted risk of pneumococcal carriage by 6% (95% CI 2-9) [17]. Few detailed data are available on contact patterns in LMICs and pneumococcal carriage, and none in a setting where PCV is being used routinely. Differences in social mixing patterns could contribute to ethnic disparities in pneumococcal carriage prevalence and disease [17].

Fiji introduced the 10-valent PCV (PCV10) in October 2012. Our previous PCV10 impact evaluation study found PCV10 carriage prevalence, three years following introduction, to be higher in indigenous Fijians (iTaukei) compared with Fijian of Indian Descent (FID), for 5–8 week olds (7.4% [95% CI 4.6-11.0] vs. 3.6 [95% CI 1.5-7.4]), 12–23 month olds (8·4% [95% CI 5·5-12·2] vs. 5·6% [95% CI 2·8-9·8]) and 2–6 year olds (12·4% [95% CI 8·9-16·8] vs. 4·4% [95% CI 2·0-8·2]) [14]. Furthermore, iTaukei are more than four times more likely to develop invasive pneumococcal disease (IPD) than FID (IRR 4.3 [95% CI 2.1-10.3]) [18]. The underlying reasons for ethnic differences in pneumococcal carriage and IPD in Fiji are unknown, but may be due to differences in host and/or environmental factors. Anecdotally, iTaukei children have more frequent social interactions, compared with FID children. A meal-time household survey in Fiji found strong assortative mixing by age and ethnicity, with higher contact rates amongst iTaukei than non-iTaukei Fijians [19]. We hypothesized that differences in contact frequency and intensity may explain ethnic differences in pneumococcal carriage and density in Fiji. This setting provides a unique opportunity to investigate ethnic differences in pneumococcal carriage and density, in a post-PCV LMIC. The aims of this study in Fiji are to (1) to determine whether the frequency and intensity of social contact differs by ethnicity; and (2) to estimate the association of contact frequency and intensity, and ethnicity, with pneumococcal carriage and density.

## 2 Materials and methods

### 2.1 Setting and source population

The study design has been published elsewhere [14]. Briefly, as part of a PCV10 impact evaluation, a cross-sectional community-based nasopharyngeal carriage survey was conducted in Suva, the capital of Fiji, and nearby villages from 13th August to 20th November 2015. PCV10 was introduced as a three dose schedule, given at 6, 10, and 14 weeks of age, with no catch-up campaign. In 2015, coverage of the first, second, and third dose of PCV10 was 82.1%, 90.3%, and 89%, respectively [20]. We used a non-probability (i.e. purposive, quota) sampling method, such that our sample matched the national iTaukei / FID population ratio (3:2), and rural / urban dweller ratio (1:1), as these factors previously were reported to be associated with pneumococcal carriage [18], [21], [22].

Participant groups were aged 5–8 weeks (young infants), 12–23 months (toddlers), 2–6 years (young children), and parents or guardians (caregivers) of child participants. Caregivers provided written informed consent for themselves and their child participants during fieldworker house visits, well-baby checks, or immunization visits at health centres. Inclusion criteria were ages as above, or caregiver status, and for non-infant participants having lived in the area for three or more months at the time of survey. Those with an axillary temperature of >37.0 °C were excluded. Young infants who had ever received a dose of PCV10 were also excluded. Some participants belonged to the same household, (defined as living, eating, and sleeping in the same dwelling) generating household clusters. The sample size (n = 500 for each participant group) was based on primary research questions regarding direct effects of PCV10 in Fiji [14]. For convenience, this study used the same sample.

This study was performed according to the protocol approved by the Fiji National Research Ethics Review Committee (Ethics Number 2101228), and the University of Melbourne Human Research Ethics Sub-Committee (Ethics ID 1238212. 4).

### 2.2 Data and sample collection

Study staff administered a demographic questionnaire, including self-reported ethnicity (iTaukei, FID, or other), age, residential location (rural/urban), antibiotic use in the fortnight preceding survey, exposure to household cigarette smoke, weekly family income, and number of people, children, and children under five years living in the household. Study team members recorded PCV10 vaccination status from written immunization records. We defined symptoms of upper respiratory tract infection (URTI) as presence of any of the following: coryzal symptoms, rhinorrhoea, cough, ear discharge, or sneezing.

After completing the demographic survey, study staff and caregivers discussed details of a social contact questionnaire, modified to the Fijian context [15]. Staff gained permission to contact the caregiver the following day to carry out the contact survey. An experienced study nurse interviewed caregivers by telephone 24 h after the nasopharyngeal swab was taken, and recorded contact details for them and each of their child participants over the previous 24 h. Contact intensity (physical or non-physical); location (home, work, transport, leisure, other); general regularity (daily, once or twice per week once or twice per month, less than monthly, never before); and duration (<5 min, 5–14 min, 15–59 min, 1–4 h, ≥4 h), and age of contacts were recorded. Childcare is rare in Fiji, and therefore childcare contacts were not recorded. As school-age children were not recruited, school-based contacts were not recorded.

We measured contact intensity as physical or non-physical [15]. For all age groups, physical contact was defined as skin-to-skin contact. Non-physical contact was defined as all other contact in the physical presence of another person, without skin-to-skin contact. There was no prescribed minimum distance to qualify as a non-physical interaction. Contact persons were counted once, and recorded as physical or non-physical. If a contact person made both physical and non-physical contact, physical contact was recorded. For each participant, contact frequency (number per 24 h) was summed by intensity, location, duration, and general regularity.

Nasopharyngeal swabs were collected, processed, stored, and transported to the Murdoch Children’s Research Institute in Melbourne, Australia, in accordance with World Health Organization guidelines [23].

### 2.3 Laboratory methods

Laboratory methods have been described previously [14]. In brief, after enzymatic lysis, we extracted DNA from nasopharyngeal swabs using the MagNA Pure LC instrument (Roche, Melbourne, Australia). We detected and quantified pneumococci in swab samples using real-time quantitative-polymerase chain reaction with primers and probes targeting the *lytA* gene, and standard curve genomic DNA from a reference isolate [24]. We undertook molecular serotyping of pneumococcal positive samples using DNA microarray, as described previously with some minor modifications [25]. Specifically, we cultured samples on horse blood agar with 5 µg/ml of gentamicin. The QIACube HT instrument (Qiagen, Melbourne, Australia) was used for DNA extraction.

Overall pneumococcal carriage was defined as detection of any pneumococci, including non-encapsulated pneumococci. PCV10 carriage was defined as detection of pneumococci included in PCV10 (serotypes 1, 4, 5, 6B, 7F, 9V, 14, 18C, 19F, and 23F). Non-PCV10 carriage was defined as detection of pneumococcal serotypes not included in PCV10, including non-encapsulated pneumococci. Although PCV10 induces an antibody response to serotype 6A, and has been shown in other settings to provide cross-protection against serotype 6A IPD, we previously found no robust evidence of cross-protection against carriage of serotype 6A in the first three years post-PCV10 introduction in Fiji [14], [26], [27]. As such, we categorized serotype 6A as a non-PCV10 serotype. Density analysis was restricted to pneumococcal positive samples and was reported in genome equivalents per ml (GE/ml). Microbiologists were blinded to participant demographic and contact data.

### 2.4 Statistical analyses

Categorical variables were summarised by numbers and percentages, and compared by the *χ*^2^ test. Continuous variables were summarised by means and 95% confidence intervals (95% CI), or medians and inter-quartile ranges (IQR). Means and medians were compared using the t-test and Mann-Whitney test, respectively. Significance was set at *P* < 0.05. Only iTaukei (n = 1212) and FID (n = 802) participants were included, due to the small number of participants of “other” ethnicity (n = 6).

We created age-stratified contact matrices per ethnic group, to explore total (physical and non-physical) and physical contact patterns. Each matrix element (*m_ij_*) is the estimated mean contacts between participants in age class *j* with contact persons in age class *i*, using estimated population sizes for the age classes to reflect the age distribution of the Fijian population in 2015, and adjusted for the reciprocal nature of contacts at the population level [22], [28], [29]. Children aged 7–14 years were not recruited. However study participants reported contacts in the 7–14 year age class, allowing inference due to reciprocity. Therefore, we were able to estimate the mean number of contacts persons for hypothetical 7–14 year old participants with all other age classes, except their own. Results are reported as estimated total number of contacts per 24 h per age class (stratified by ethnic group), and the relative rates of contact per age class for iTaukei participants was compared with FID participants. Age classes were defined in line with the sampling strata: infants (<12 months), toddlers (12–23 months), young children (2–6 years), older children (7–14 years), adults (15+ years).

The associations of carriage and density with frequency of physical and non-physical contacts were analysed using univariable generalised estimating equation (GEE) models. Non-physical contact was not associated with carriage or density, so not considered for inclusion in multivariable models. Associations between contact and carriage (overall, PCV10, and non-PCV10) were examined using logistic GEE models, with results expressed as odds ratios and 95% CI. The associations of contact with density (overall, PCV10, and non-PCV10) were investigated using linear GEE models, with analyses restricted to pneumococcal carriers. Pneumococcal density was log_10_ transformed prior to analysis to remove skewness. Results are reported as mean differences in pneumococcal density (log_10_GE/ml) and 95% CI. GEE analyses used an exchangeable working correlation structure, under the assumption that household clustering had no logical ordering [30]. The frequency of contacts per age class was the primary measure of contact. Contact location, duration, and general regularity, were correlated with physical contact, and therefore excluded from further analyses.

We used a combination of *a priori* (ethnicity and residential location) and empirical (*P* < 0.05 from univariable GEE models) approaches to inform variable selection for multivariable GEE models. Additional variables adjusted for in logistic GEE models for carriage included current symptoms of URTI, participant group, poverty, sex of participant, PCV10 vaccination status, and number of people living in the household. Additional variables adjusted for in linear GEE regression models for overall and non-PCV10 density comprised residential location, current symptoms of URTI, participant group, PCV10 vaccination history, and number of people living in the household. Variables adjusted for in linear GEE regression models for PCV10 density were the same as those for overall and non-PCV10 except number of people living in the household. Interaction between ethnicity and contact was considered but not included in the final models, as the relationship between contact and carriage was the similar for the ethnic groups.

## 3 Results

We enrolled 2014 participants, including 496 young infants, 498 toddlers, 510 young children, and 510 of their caregivers. The median ages of iTaukei and FID caregivers was 33 years (IQR 26-49) and 31 years (IQR 25-40), respectively). iTaukei and FID participants had similar distributions of sex, participant group, residential location, antibiotic use in the fortnight preceding survey, exposure to household cigarette smoke, poverty, and PCV10 vaccination status (Table 1). PCV10 vaccination rates were 30.9% for all iTaukei and 29.7% for all FID participants, and 98.7% and 97.1% in PCV10 age-eligible children, respectively. Compared with FID participants, a greater percentage of iTaukei participants reported symptoms of URTI. iTaukei participants lived in households with an average of 2.4 more people (1.5 more children under 18 years, and 0.7 more children under five years), compared with FID participants. Twenty-two participant samples were excluded from analysis due to insufficient volume, sample loss, labelling errors, or other technical issues. Pneumococcal carriage (overall, PCV10 and non-PCV10) prevalence, but not density, was greater among iTaukei, compared with FID participants (Table 1).

**Table 1:** Characteristics of participants in a cross-sectional carriage and contact survey in Fiji, 2015.

Characteristics	iTaukei	Fijian of Indian Descent	*P*^b^
	(n = 1212^a^)	(n = 802^a^)	
Male, n (%)	511 (42.2)	319 (39.8)	0.29

Participant group, n (%)			0.95
Young infants	303 (25.0)	193 (24.1)	
Toddlers	301 (24.8)	197 (24.6)	
Young children	303 (25.0)	207 (25.8)	
Caregivers	305 (25.2)	205 (25.5)	

Residence, n (%)			0.29
Urban	641 (52.9)	405 (50.5)	
Rural	571 (47.1)	397 (49.5)	

Symptoms of URTI, n (%)	393 (32.4)	163 (20.3)	<0.01

Antibiotics in past fortnight, n (%)	43 (3.6)	18 (2.2)	0.09

Exposure to household cigarette smoke, n (%)	658 (54.3)	429 (53.5)	0.73

Poverty^c^, n (%)	631 (52.1)	426 (53.1)	0.64

Household size, mean (95% CI)			
All ages	8.1 (7.9, 8.3)	5.7 (5.6, 5.8)	<0.01
Aged <18 years	3.8 (3.7, 4.0)	2.3 (2.2, 2.4)	<0.01
Aged <5 years	2.1 (2.1, 2.2)	1.4 (1.4, 1.5)	<0.01
PCV10 vaccinated^d^, n %)	375 (30.9)	238 (29.7)	0.55

Pneumococcal carriage, n/N; % (95% CI)			
Overall^e^	538/1195; 45.0 (42.2, 47.9)	108/797; 13.5 (11.2, 16.1)	<0.01
PCV10 serotypes^f^	85/1182; 9.0 (7.5, 10.8)	29/796; 3.6 (2.4, 5.2)	<0.01
Non-PCV10 serotypes^g^	476/1182; 38.3 (35.5, 41.2)	80/796; 10.0 (8.5, 12.3)	<0.01
Pneumococcal density^h^ n; median (IQR)	538; 4.9 (4.0, 5.7)	108; 4.7 (3.9, 5.5)	0.16

URTI: upper respiratory tract infection.

a Unless otherwise indicated.

b χ^2^ test and *t*-tests compared categorical and continuous variables, respectively.

c Family income <FJ$175/wk. [23].

d Received at least two doses of PCV10.

e Any pneumococci, including non-encapsulated pneumococci.

f Pneumococci included in PCV10 (serotypes 1, 4, 5, 6B, 7F, 9V, 14, 18C, 19F, and 23F).

g Pneumococcal serotypes not included in PCV10, nor serotype 6A, including non-encapsulated pneumococci.

h Includes only participants who were pneumococcal carriers.

Contact measures were collected from participants from 1035 households, who reported 12,937 contacts. Most contacts occurred at home (96.6%), occurred daily (96.1%), and lasted longer than four hours (96.2%). The mean frequency of physical contact was higher compared with that of non-physical contact (Table 2). iTaukei participants reported an average of 2.4 more total contacts, 1.4 more physical contacts, and 1.0 more non-physical contact compared with FID (Table 2). Similarly, iTaukei participants reported more contacts made at home, of longer duration, and made on a daily basis, compared with FID participants.

**Table 2:** Mean number (95% confidence interval) of contacts per 24 h by: intensity, location, general regularity, and duration, reported by study participants in a pneumococcal carriage and contact survey in Fiji, stratified by ethnicity (n = 2014).

Contact measures	Total (n = 2014)		iTaukei (n = 1212)		Fijian of Indian Descent (n = 802)		*P*^a^
	Mean	95% CI	Mean	95% CI	Mean	95% CI	
Total contacts	6.40	6.24, 6.55	7.36	7.14, 7.59	4.94	4.78, 5.09	<0.01

Intensity							
Non-physical	1.43	1.32, 1.53	1.82	1.67, 1.97	0.83	0.73, 0.94	<0.01
Physical	4.97	4.87, 5.07	5.55	5.40, 5.69	4.10	3.98, 4.23	<0.01

Location							
Leisure activities	0.22	0.18, 0.26	0.23	0.18, 0.29	0.19	0.14, 0.24	0.28
Home	6.18	6.02, 6.33	7.13	6.91, 7.35	4.74	4.59, 4.89	<0.01

General regularity							
Less than once per month	0.04	0.02, 0.07	0.04	0.00, 0.07	0.05	0.01, 0.09	0.62
1–2 times per month	0.06	0.04, 0.08	0.06	0.04, 0.09	0.06	0.03, 0.09	0.76
1–2 times per week	0.14	0.11, 0.17	0.16	0.11, 0.20	0.12	0.08, 0.15	0.20
Daily	6.15	6.00, 6.30	7.10	6.88, 7.32	4.71	4.57, 4.85	<0.01

Duration							
5–14 min	0.02	0.01, 0.03	0.03	0.01, 0.04	0.02	0.01, 0.03	0.50
15–59 min	0.05	0.03, 0.07	0.05	0.03, 0.08	0.05	0.02, 0.07	0.62
1–4 h	0.16	0.13, 0.20	0.20	0.14, 0.26	0.11	0.07, 0.15	0.03
>4 h	6.15	6.00, 6.31	7.08	6.86, 7.29	4.76	4.61, 4.90	<0.01

a iTaukei compared with Fijian of Indian Descent by *t*-test.

**Fig. 1:**
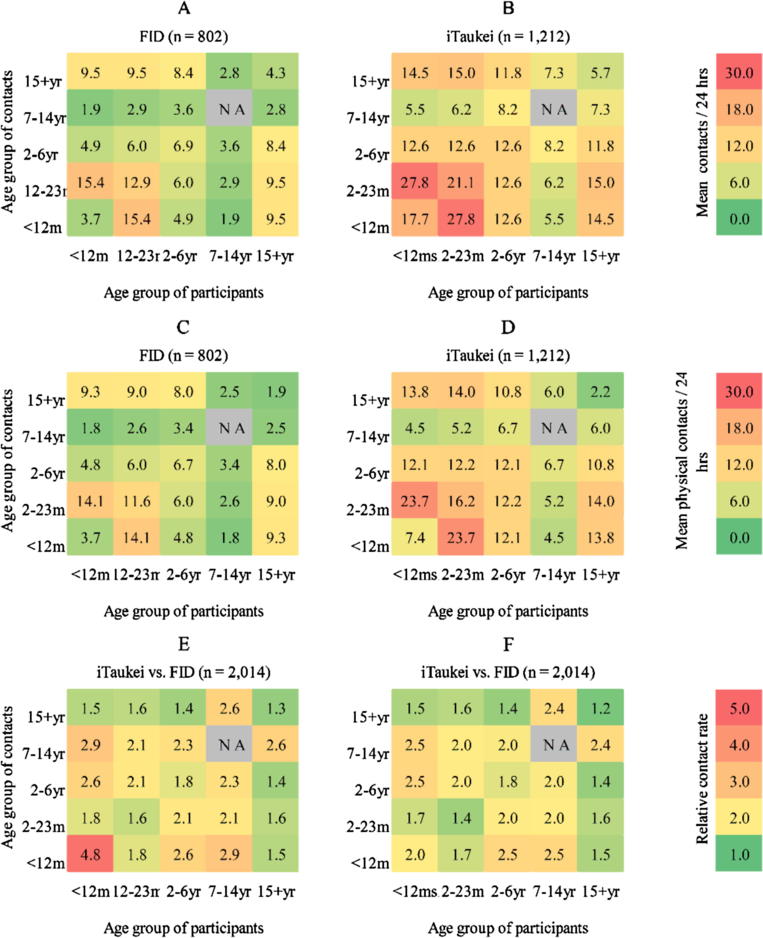
Age stratified contact matrices by ethnic group. (A) Mean estimated total contacts per 24 h for Fijians of Indian Descent (FID); (B) Mean estimated total contacts per 24 h for iTaukei; (C) Mean estimated physical contacts for FID; (D) Mean estimated physical contacts for iTaukei (E); Relative rate of total contact, iTaukei compared with FID; (F) Relative rate of physical contact, iTaukei compared with FID.

Fig. 1 shows age-stratified estimates of mean total and physical contacts per 24 h for FID (Fig. 1A and C) and iTaukei (Fig. 1B and 1D) participants. Total and physical contact matrices are similar per ethnic group, reflecting that most contacts were physical. iTaukei participants had higher estimated mean total and physical contacts compared with FID participants for all participant groups. For both ethnic groups, the highest estimates of total and physical contacts were between toddlers, and between toddlers and infants. Fewest contacts were made between older children and infants in both ethnic groups. Caregiver participants of both ethnicities had the highest reported number of total and physical contacts with young infants, toddlers, and young children. Compared with other age classes, and with FID counterparts, iTaukei young infant participants had the highest relative rate of total contact with other infants per 24 h (Fig. 1E). However, the highest relative rate of estimated physical contact was between iTaukei infants and iTaukei young and older children (Fig. 1F), compared with FID age based counterparts.

Table 3, Table 4 show the unadjusted and adjusted odds ratios of overall and PCV10 pneumococcal carriage in association with physical contact with infants, toddlers, young children, older children, and adults. Results for non-PCV10 carriage are shown in Supplementary Table 1. There was evidence of an association between overall carriage and physical contact with toddlers, young, and older children (Table 3). Frequency of physical contact with older children increased odds of PCV10 carriage (Table 4). There was some evidence of an association between non-PCV10 carriage and frequency of physical contact with toddlers and young children (Supplementary Table 1). iTaukei ethnicity was associated with higher odds of all types of carriage. Symptoms of URTI increased odds of overall, and non-PCV10 carriage. Compared with toddlers, caregivers had lower odds of carriage (overall, PCV10, and non-PCV10).

**Table 3:** Unadjusted and adjusted odds ratios showing the association of frequency of physical contact by age class with overall pneumococcal nasopharyngeal carriage in a cross-sectional carriage and contact survey, Fiji, 2015 (n = 1993)^a^.

Covariate	Unadjusted odds ratio	95% CI	*P*	Adjusted odds ratio^b^	95% CI	*P*
Number of physical contacts per 24 h with:						
Infants	0.95	0.77, 1.16	0. 62	0.94	0.73, 1.22	0.65
Toddlers	1.22	1.03, 1.46	0.02	1.34	1.07, 1.68	<0.01
Young children	0.98	0.89, 1.09	0.73	1.12	0.98, 1.28	0.11
Older children	1.24	1.13, 1.37	<0.01	1.10	0.98, 1.23	0.10
Adults	1.26	1.19, 1.33	<0.01	0.99	0.92, 1.08	0.88

Fijian of Indian Descent	*ref*	*ref*		*ref*	*ref*	
iTaukei	5.21	4.10, 6.63	<0.01	5.16	3.95, 6.75	<0.01

Urban residence	*ref*	*ref*		*ref*	*ref*	
Rural residence	0.81	0.66, 1.00	0.05	0.84	0.67, 1.06	0.14
Symptoms of URTI	2.53	2.06, 3.11	<0.01	2.00	1.58, 2.55	<0.01
Household cigarette exposure	0.94	0.77, 1.15	0.56			
Poverty^c^	1.01	0.82, 1.24	0.93	1.09	0.87, 1.38	0. 45

Participant group						
Toddlers	*ref*	*ref*		*ref*	*ref*	
Young infants	0.73	0.57, 0.94		0.70	0.40, 1.22	
Young children	1.01	0.78, 1.30	<0.01	0.98	0.62, 1.55	<0.01
Caregivers	0.11	0.07, 0.16		0.08	0.04, 0.15	

Male	*ref*	*ref*		*ref*	*ref*	
Female	0.57	0.47, 0.69	<0.01	1.00	0.80, 1.25	0.98
PCV10 vaccinated^d^	2.04	1.68, 2.49	<0.01	0.97	0.61, 1.55	0.90
Antibiotics in past fortnight	0.92	0.53, 1.59	0.77			
Number of people living in the household	1.09	1.06, 1.12	<0.01	1.00	0.95, 1.05	0.92

URTI: upper respiratory tract infection.

a Any pneumococci, including non-encapsulated lineages.

b Covariates adjusted for were residential location, current symptoms of upper respiratory tract infection, poverty, participant group, sex, PCV10 vaccination status, and number of people living in the household.

c Family income <FJ$175/wk. [23].

d At least two doses of PCV10.

**Table 4:** Unadjusted and adjusted odds ratios showing the association of frequency of physical contact by age class with PCV10 pneumococcal nasopharyngeal carriage in a cross-sectional carriage and contact survey, Fiji, 2015 (n = 1978)^a^.

Covariate	Unadjusted odds ratio	95% CI	*P*	Adjusted odds ratio^b^	95% CI	*P*
Number of physical contacts per 24 h with:						
Infants	0.96	0.63, 1.45	0.83	1.02	0.65, 1.62	0.92
Toddlers	1.20	0.86, 1.68	0.28	1.27	0.87, 1.87	0.22
Young children	0.85	0.67, 1.06	0.15	0.94	0.73, 1.21	0.63
Older children	1.31	1.12, 1.53	<0.01	1.28	1.08, 1.53	<0.01
Adults	1.17	1.07, 1.29	<0.01	1.06	0.92, 1.21	0.44

Fijian of Indian Descent	*ref*	*ref*		*ref*	*ref*	
iTaukei	1.96	1.25, 3.08	<0.01	1.73	1.06, 2.82	0.03

Urban residence	*ref*	*ref*		*ref*	*ref*	
Rural residence	1.18	0.79, 1.77	0.42	1.23	0.82, 1.85	0.32
Symptoms of URTI	1.80	1.21, 2.68	0.01	1.53	1.01, 2.32	0.04
Household cigarette exposure	0.82	0.55, 1.22	0.33			
Poverty^c^	0.91	0.61, 1.36	0.64	0.97	0.64, 1.47	0.89

Participant group						
Toddlers	*ref*	*ref*		*ref*	*ref*	
Young infants	0.81	0.46, 1.35		0.49	0.18, 1.31	
Young children	1.30	0.82, 2.07	<0.01	0.86	0.37, 1.98	<0.01
Caregivers	0.09	0.03, 0.28		0.07	0.02, 0.28	

Male	*ref*	*ref*		*ref*	*ref*	
Female	0.59	0.41, 0.88	<0.01	0.91	0.61, 1.35	0.65
PCV10 vaccinated^d^	1.42	0.96, 2.10	0.08	0.67	0.32, 1.44	0.23
Antibiotics in past fortnight	0.27	0.04, 1.96	0.20			
Number of people in the household	1.05	1.00, 1.10	0.07	0.97	0.89, 1.06	0.49

URTI: upper respiratory tract infection.

a Pneumococcal serotypes included in PCV10 (serotypes 1, 4, 5, 6B, 7F, 9V, 14, 18C, 19F, and 23F).

b Covariates adjusted for were ethnicity, residential location, current symptoms of upper respiratory tract infection, poverty, participant group, sex, PCV10 vaccination status, and number of people living in the household.

c Family income <FJ$175/wk. [23].

d At least two doses of PCV10.

Among pneumococcal carriers (646) there were no associations between pneumococcal density (overall, PCV10, or non-PCV10) and frequency of physical contacts, or ethnicity (Table 5 and Supplementary Tables 2–3). Higher mean overall pneumococcal density was associated with young children compared with toddlers, having URTI symptoms, and a rural residential location. (Table 5). Higher mean density of both PCV10 and non-PCV10s were found in association with URTI symptoms, and young children compared with toddlers (Supplementary Tables 2 and 3). Mean density of PCV10 pneumococci was higher in association with a rural compared with an urban residence (Supplementary Tables 2 and 3).

**Table 5:** Unadjusted and adjusted mean difference showing the association of frequency of physical contact by age class with overall pneumococcal nasopharyngeal density, GE/ml log_10_ scale, in a cross-sectional carriage and contact survey, Fiji, 2015 (n = 646)^a^.

Covariate	Unadjusted mean difference	95% CI	*P*	Adjusted mean difference^b^	95% CI	*P*
Number of physical contacts per 24 h with:						
Infants	0.24	0.06, 0.41	0.01	0.08	−0.01, 0.26	0.38
Toddlers	−0.05	−0.20, 0.10	0.545	−0.06	−0.21, 0.10	0.46
Young children	0.10	0.02, 0.18	0.02	0.00	−0.09, 0.09	0.98
Older children	0.05	−0.03, 0.13	0.21	−0.01	−0.09, 0.06	0.72
Adults	0.00	−0.05, 0.05	1.00	−0.01	−0.06, 0.05	0.84

Fijian of Indian Descent	*ref*	*ref*		*ref*	*ref*	
iTaukei	0.17	−0.06, 0.41	0.15	0.11	−0.11, 0.33	0.33

Urban residence	*ref*	*ref*		*ref*	*ref*	
Rural residence	0.19	0.00, 0.37	0.04	0.22	0.06, 0.40	<0.01
Symptoms of URTI	0.40	0.23, 0.58	<0.01	0.32	0.15, 0.49	<0.01
Household cigarette exposure	0.05	−0.13, 0.23	0.59			
Poverty^c^	−0.09	−0.27, 0.09	0.33			

Participant group						
Toddlers	*ref*	*ref*		*ref*	*ref*	
Young infants	−0.26	−0.47, −0.05		−0.27	−0.66, 0.13	
Young children	0.65	0.46, 0.85	<0.01	0.58	0.26, 0.90	<0.01
Caregivers	0.41	0.05, 0.76		0.34	−0.14, 0.83	

Male	*ref*	*ref*				
Female	−0.05	−0.22, 0.12	0.58			
PCV10 vaccinated^d^	−0.07	−0.24, 0.10	0.44	−0.05	−0.38, 0.28	0.77
Antibiotics in past fortnight	0.31	−0.19, 0.81	0.22			
Number of people living in the household	0.03	0.01, 0.05	0.01	0.02	−0.01, 0.05	0.30

URTI: upper respiratory tract infection.

a Only includes participants who were pneumococcal carriers.

b Covariates adjusted for physical contact with infants, toddlers, young children, older children, adults; ethnicity, residential location, symptoms of upper respiratory tract infection, participant group, sex, PCV10 vaccination status, and number of people living in the household.

c Family income <FJ$175/wk. [23].

d At least two doses of PCV10.

## 4 Discussion

This is the first study to investigate the association between physical contact, ethnicity, and pneumococcal carriage and density in a post-PCV setting. The iTaukei population had higher pneumococcal carriage prevalence compared with their FID counterparts, despite similar poverty levels. Contact frequency and intensity varied by ethnicity. Frequency of physical, but not non-physical, contact was associated with pneumococcal carriage. Post-PCV10 introduction, physical contact with older children was associated positively with PCV10 carriage. These findings lend support to the hypothesis that close physical contact with older unvaccinated children is important for pneumococcal transmission in the post-PCV introduction and may have implications for pneumococcal disease control, particularly in settings with large household and classroom sizes. PCV10 and non-PCV10 pneumococcal density were not associated with ethnicity, or contact intensity or frequency.

Most contacts were physical, occurred at home, and had a long duration (4+ hours). This likely reflects that participants were either too young to attend school, or were caregivers of pre-school children. In this setting, sharing sleeping spaces are common, which likely drives the mean duration of contact. Previous contact frequency studies have been conducted in varied contexts, including Europe, South-East Asia, and sub-Saharan Africa [15], [17], [19], [31], [32], [33], [34]. Mossong et al. conducted a population-based prospective survey of mixing patterns in eight European countries, involving 7290 participants and 97,904 reported contacts (POLYMOD study) [15]. Horby et al. conducted a household-based cohort study in a semi-rural community on the Red River Delta of North Vietnam involving 865 participants and 6675 reported contacts [31]. Le Polain de Waroux et al. conducted a cluster, age-stratified, cross-sectional community-based survey of contact patterns in rural Uganda involving 566 participants from Sheema North, rural Uganda [17]. Direct comparison of findings from these studies, and others, is hampered by differing settings, participant age categories, and survey methods. However, iTaukei participants reported a similar number of contacts per 24 h (7.36) compared with participants in rural Uganda (7) and Vietnam (7.7), while FID participants reported fewer contacts (4.93) [17], [31]. Participants in the POLYMOD study reported a higher mean number of contacts per 24 h, compared with iTaukei and FID participants [15]. In our study, the highest contact rates were between toddlers with infants, and with other toddlers, which may reflect children sharing sleeping spaces. There was some evidence in both ethnic groups, of age assortative mixing, similar to previous findings from Fiji, Europe, Japan, rural Uganda, Vietnam, and Zimbabwe [15], [17], [19], [32], [35]. This may be why young children are important transmitters to other young children of respiratory pathogens in general, and for pneumococci in particular.

The greater contact frequency and intensity rates in iTaukei participants, compared with FID, likely reflects the greater number of people living in iTaukei households. These findings reflect those of Watson et al. who conducted a cross-sectional mealtime contacts survey in Fiji (n = 1814) and reported age and ethnic assortative mixing, with higher rates of contact between iTaukei than FID [19]. Previous studies have reported a possible association between larger household sizes and increased contact frequency and intensity. Mossong et al. reported that larger household size was associated with greater contact number, and that 75% of contacts made at home were physical [15]. An Australian household structure study found that on average, Indigenous Australian pre-school, school-age, and adult household members had 25, 15–20, and ~4 times greater household contacts, respectively, compared with non-indigenous counterparts, largely reflecting relative household sizes [36]. A household structure and contact survey in Japan found households of three or more people reported greater mean contacts per 24 h compared with households with fewer members [32]. In contrast, Horby et al. found no association between the total number of recorded contacts and household size, and in rural Uganda, only moderate correlation (R = 0.61) was found between the number of physical and household contacts [17], [31].

Importantly, we found physical contact with older children was associated with PCV10 carriage. In the early post-PCV era, unvaccinated older children may be a PCV10 reservoir, as these children were age-ineligible for PCV10 vaccination and herd effects may not yet have manifested. Overall and non-PCV10 carriage were associated with physical contact with toddlers and young children, consistent with the literature [6]. Our findings are consistent with a longitudinal cohort study of the Navajo Nation and White Mountain Apache American Indian Tribes, in which a probability transmission model found contact with toddlers and older children was associated with pneumococcal transmission and acquisition in children of similar and younger age groups [7]. In addition, they found that pneumococcal transmission could also be from infants to people aged over fifteen years [7]. Most of the caregivers in our study were older, therefore we did not observe this in our study. A post-PCV7 outbreak of invasive pneumococcal disease, mainly in adults, from indigenous communities from Central Australia was caused by pneumococcal serotype 1 [37]. In those communities, researchers found that serotype 1 carriers were children aged 5–8 years, suggesting older children may be a source of pneumococcal carriage transmission [37].

Further, a recent study in Israel found that vaccine-type carriage did not decline in children over 2 years old until the cohorts of children who were vaccinated as infants entered those older age group categories, despite a catch up campaign to two years of age [8]. We found a relatively low rate of contact between older children and infants, suggesting pneumococcal transmission from older children to infants too young to be vaccinated may not be clinically relevant. Although we have no carriage data for older children, our broader PCV10 impact study found an annual decline in the PCV10 carriage prevalence in young children after the introduction of PCV10 [14]. This suggests that PCV10 carriage prevalence in older children is unlikely to have increased post-PCV10 in *absolute* terms, but may have become important *relative* to the usual transmitters - the now vaccinated toddlers [14]. Carriage of pneumococci in the post-PCV era requires ongoing monitoring, particularly in settings with large household /school classroom sizes, as school-age children may be an important source of ongoing transmission.

This is the first study to investigate the relationship between physical contact and ethnicity on pneumococcal density, finding no association. We also observed no association between PCV10 vaccination and pneumococcal density (overall, PCV10, or non-PCV10). However, young age, symptoms of URTI, and rural residential location were associated with higher mean pneumococcal density. Our findings are consistent with several studies that found increased pneumococcal density in association with URTI symptoms [4], [38], [39], [40]. Studies comparable to ours, with fully quantitative methods to define pneumococcal density, have heterogeneous observations with regard to the impact of PCV on pneumococcal density. Sigaque et al. investigated the impact of PCV10 vaccination on density of serotypes 11A, 19A, and 19F in children aged 6 weeks to 59 months in Mozambique, and observed no association [41]. In Lao PDR following PCV13 introduction, density of both PCV13 and non-PCV13 pneumococci increased in 12–23 month old children [42]. Hanke et al. observed that in 125 Peruvian Andean children, PCV7 vaccination did not impact density of PCV7 pneumococci, but increased density of non-PCV7 pneumococci [43]. These differing findings reflect the complex relationship between host, viral, and bacterial organisms.

There were a number of limitations to our study. Firstly, our findings may not be generalizable to other areas of Fiji as participants were a population of Fijians living in the Central Division, Suva. Reported contact patterns may not be representative of all contacts. Our study was conducted over a four-month period, and on weekdays only. Weekend contact patterns, particularly those involving communal church services on Sundays, likely differ by ethnicity [22]. Sampling bias may have occurred due to our non-random sampling strategy. However, we ensured the sample was representative of ethnic and rural/urban demographics of Fiji through purposive quota sampling [18], [21]. The contact survey was prone to non-differential misclassification bias, as the behaviour of the survey subject was reported, but not that of their contacts [44]. Caregivers responding to contact survey questions may not have recalled all contacts [28]. This may be particularly the case when participants were asked to distinguish between physical and non-physical contacts, and potentially more so for infants. We attempted to minimise this recall bias by discussing the contact survey with caregivers one day before contact survey administration, as the accuracy of reporting is known to improve when individuals are primed to report contacts in advance [16]. This was a cross-sectional study and therefore provides no temporal association between contact, and pneumococcal carriage or density. The collection of nasopharyngeal swabs occurred at one time point, so we cannot infer pneumococcal transmission but rather an association. Contact was measured for the 24 h after swab collection, and may not necessarily be the same as contact behaviour preceding swab collection or colonization. Logistic and pragmatic constraints of the study permitted contact to be measured for 24 h, and our contact findings may not be representative of daily contact.

Results regarding the association of ethnicity and physical contact frequency by age group with PCV10 density should be interpreted with caution due to small sample sizes. We had no data on general morbidity, nutritional status, and exposure to household smoke from cooking fuels, or respiratory viruses, which may be important with regard to pneumococcal carriage [21], [45], [46], [47], [48]. Similarly, we had no data on vaccination status of contacts, which may have helped explain the importance of different age classes for indirection protection. Finally, analysis of the association between contact frequency and intensity and serotype-specific density was not possible, due to small numbers of participants carrying individual serotypes.

Fiji is an early adopter of PCV. Our unique study provides important insights into other Asia Pacific settings that will introduce PCV in the future. Our study demonstrates that contact frequency and intensity differs by ethnicity in Fiji. We found that ethnicity and frequency of intergenerational physical contact are associated with pneumococcal carriage. However, our data suggest that contact patterns alone are insufficient to explain ethnic differences in carriage burden, which remain strong after adjustment. Our study identified the potential key role of older unvaccinated children in association with vaccine serotypes post-PCV introduction, previously an age group considered less important to pneumococcal transmission.

In conclusion, we observed that differences in physical contact frequency were associated with pneumococcal carriage, but do not explain ethnic differences in pneumococcal nasopharyngeal carriage in Fiji. These findings may have implications for pneumococcal disease control in early post-PCV settings in LMICs countries, particularly for populations with large household sizes, because such settings increase rates of physical contact. This study provides evidence that older, unvaccinated, children may become important in transmission of pneumococci, particularly PCV pneumococci, in post-PCV settings. With movement towards reduced dose PCV schedules, monitoring remaining sources of PCV serotype transmission closely will be necessary for pneumococcal disease control [49]. Follow up studies are needed to investigate whether herd immunity eventually eliminates PCV serotype circulation, or whether school-age children remain a reservoir for PCV serotype transmission in this setting.

## Declaration of Competing Interest

The authors declare that they have no known competing financial interests or personal relationships that could have appeared to influence the work reported in this paper.
