# Supplementary material for: Associations between ethnicity, social contact, and pneumococcal carriage three years post-PCV10 in Fiji
Source: Vaccine. 2020 Jan 10;38(2):202–11. doi: 10.1016/j.vaccine.2019.10.030 (PMC6964150; doi:10.1016/j.vaccine.2019.10.030)
Supplement: Supplementary data 1 [file mmc1.docx]

**Supplementary Table 1:** Unadjusted and adjusted odds ratios showing the association of frequency of physical contact by age class with non-PCV10 pneumococcal nasopharyngeal carriage in a cross-sectional carriage and contact survey, Fiji, 2015 (n=1,978)^a^

| Covariate | Unadjusted odds ratio | 95% CI | *P* | Adjusted odds ratio ^b^ | 95% CI | *P* |
| --- | --- | --- | --- | --- | --- | --- |
| Number of physical contacts per 24 hours with: |  |  |  |  |  |  |
| Infants | 0.97 | 0.78, 1.21 | 0.81 | 0.96 | 0.74, 1.25 | 0.76 |
| Toddlers | 1.18 | 0.98, 1.42 | 0.08 | 1.26 | 1.00, 1.58 | 0.05 |
| Young children | 1.01 | 0.91, 1.12 | 0.87 | 1.13 | 0.99, 1.29 | 0.08 |
| Older children | 1.20 | 1.09, 1.33 | <0.01 | 1.05 | 0.94, 1.18 | 0.38 |
| Adults | 1.23 | 1.16, 1.30 | <0.01 | 0.98 | 0.90, 1.06 | 0.57 |
| Fijian of Indian Descent | *ref* | *ref* |  | *ref* | *ref* |  |
| iTaukei | 6.07 | 4.63, 7.96 | <0.01 | 5.98 | 4.47, 8.00 | <0.01 |
| Urban residence | *ref* | *ref* |  | *ref* | *ref* |  |
| Rural residence | 0.80 | 0.64, 0.99 | 0.01 | 0.83 | 0.66, 1.06 | 0.13 |
| Symptoms of URTI | 2.40 | 1.94, 2.97 | <0.01 | 1.85 | 1.45, 2.36 | <0.01 |
| Household cigarette exposure | 1.02 | 0.82, 1.23 | 0.89 |  |  |  |
| Poverty^c^ | 1.03 | 0.83, 1.28 | 0.79 | 1.12 | 0.88, 1.42 | 0.34 |
| Participant group |  |  |  |  |  |  |
| Toddlers | *ref* | *ref* |  | *ref* | *ref* |  |
| Young infants | 0.72 | 0.55, 0.94 |  | 0.71 | 0.40, 1.24 |  |
| Young children | 0.97 | 0.75, 1.25 | <0.01 | 0.94 | 0.59, 1.50 | <0.01 |
| Caregivers | 0.11 | 0.08, 0.17 |  | 0.08 | 0.04, 0.16 |  |
| Male | *ref* | *ref* |  | *ref* | *ref* |  |
| Female | 0.57 | 0.47, 0.69 | <0.01 | 0.97 | 0.77, 1.22 | 0.90 |
| PCV10 vaccinated^d^ | 2.01 | 1.64, 2.46 | <0.01 | 0.96 | 0.60, 1.56 | 0.90 |
| Antibiotics in past fortnight | 1.04 | 0.59, 1.81 | 0.90 |  |  |  |
| Number of people living in the household | 1.09 | 1.06, 1.13 | <0.01 | 1.00 | 0.95, 1.05 | 0.95 |

URTI: upper respiratory tract infection; ^a^ Pneumococcal serotypes non included in PCV10, including non-encapsulated lineages; ^b^ Covariates adjusted for were ethnicity, residential location, current symptoms of upper respiratory tract infection, poverty, participant group, sex, PCV10 vaccination status, and number of people living in the household; ^c^ Family income <FJ$175/wk. [23]; ^d^ At least two doses of PCV10
