# Supplementary material for: Associations between ethnicity, social contact, and pneumococcal carriage three years post-PCV10 in Fiji
Source: Vaccine. 2020 Jan 10;38(2):202–11. doi: 10.1016/j.vaccine.2019.10.030 (PMC6964150; doi:10.1016/j.vaccine.2019.10.030)
Supplement: Supplementary data 2 [file mmc2.docx]

**Supplementary Table 2:** Unadjusted and adjusted mean difference showing the association of frequency of physical contact by age class with PCV10 pneumococcal nasopharyngeal density, GE/ml log_10_ scale, in a cross-sectional carriage and contact survey, Fiji, 2015 (n=141)^a^

| Covariate | Unadjusted mean difference | 95% CI | *P* | Adjusted mean difference^b^ | 95% CI | *P* |
| --- | --- | --- | --- | --- | --- | --- |
| Number of physical contacts per 24 hours with: |  |  |  |  |  |  |
| Infants | -0.12 | -0.36, 0.60 | 0.63 | 0.13 | -0.33, 0.60 | 0.57 |
| Toddlers | -0.36 | -0.73, 0.01 | 0.06 | -0.19 | -0.52, 0.14 | 0.26 |
| Young children | 0.08 | -0.15, 0.31 | 0.49 | 0.05 | -0.18, 0.27 | 0.68 |
| Older children | 0.13 | -0.05, 0.32 | 0.16 | 0.08 | -0.10, 0.26 | 0.39 |
| Adults | -0.02 | -0.12, 0.07 | 0.64 | 0.02 | -0.06, 0.10 | 0.57 |
| Fijian of Indian Descent | *ref* | *ref* |  | *ref* | *ref* |  |
| iTaukei | 0.30 | -0.19, 0.80 | 0.23 | 0.26 | -0.22, 0.75 | 0.28 |
| Urban residence | *ref* | *ref* |  | *ref* | *ref* |  |
| Rural residence | 0.57 | 0.15, 0.99 | <0.01 | 0.55 | 0.17, 0.93 | <0.01 |
| Symptoms of URTI | 0.64 | 0.22, 1.06 | <0.01 | 0.63 | 0.23, 1.03 | <0.01 |
| Household cigarette exposure | -0.04 | -0.48, 0.40 | 0.85 |  |  |  |
| Poverty ^c^ | -0.05 | -0.49, 0.39 | 0.83 |  |  |  |
| Participant group |  |  |  |  |  |  |
| Toddlers | *ref* | *ref* |  | *ref* | *ref* |  |
| Young infants | 0.05 | -0.51, 0.61 |  | 0.97 | -0.10, 2.04 |  |
| Young children | 0.55 | 0.05, 1.05 | 0.01 | 1.09 | 0.16, 2.01 | 0.07 |
| Caregivers | 0.68 | -0.47, 1.83 |  | 1.67 | 0.25, 3.09 |  |
| Male | *ref* | *ref* |  |  |  |  |
| Female | 0.10 | -0.33, 0.53 | 0.65 |  |  |  |
| PCV10 vaccinated ^d^ | -0.06 | -0.50, 0.38 | 0.80 | 0.95 | -0.03, 1.73 | 0.06 |
| Antibiotics in past fortnight | -0.79 | -2.95, 1.36 | 0.47 |  |  |  |
| Number of people living in the household | 0.03 | -0.03, 0.09 | 0.32 |  |  |  |

URTI: upper respiratory tract infection; ^a^ Only includes participants who were carriers of pneumococci included in PCV10 (serotypes 1, 4, 5, 6B, 7F, 9V, 14, 18C, 19F, and 23F); ^b^ Covariates adjusted for physical contact with infants, toddlers, young children, older children, adults; ethnicity, residential location, symptoms of upper respiratory tract infection, participant group, sex, and PCV10 vaccination status; ^c^ Family income <FJ$175/wk. [23]; ^d^ At least two doses of PCV10
