# Supplementary material for: Associations between ethnicity, social contact, and pneumococcal carriage three years post-PCV10 in Fiji
Source: Vaccine. 2020 Jan 10;38(2):202–11. doi: 10.1016/j.vaccine.2019.10.030 (PMC6964150; doi:10.1016/j.vaccine.2019.10.030)
Supplement: Supplementary data 3 [file mmc3.docx]

**Supplementary Table 3:** Unadjusted and adjusted mean difference showing the association of frequency of physical contact by age class with non-PCV10 pneumococcal nasopharyngeal density, GE/ml log_10_ scale, in a cross-sectional carriage and contact survey, Fiji, 2015 (n=526) ^a^

| Covariate | Unadjusted mean difference | 95% CI | *P* | Adjusted mean difference^b^ | 95% CI | *P* |
| --- | --- | --- | --- | --- | --- | --- |
| Number of physical contacts per 24 hours with: |  |  |  |  |  |  |
| Infants | 0.22 | 0.03, 0.41 | 0.02 | 0.07 | -0.11, 0.26 | 0.43 |
| Toddlers | 0.00 | -0.16, 0.16 | 0.98 | -0.03 | -0.19, 0.14 | 0.75 |
| Young children | 0.09 | 0.00, 0.17 | 0.03 | 0.01 | -0.08, 0.10 | 0.86 |
| Older children | 0.02 | -0.06, 0.10 | 0.65 | -0.04 | -0.12, 0.04 | 0.36 |
| Adults | 0.01 | -0.05, 0.07 | 0.78 | 0.01 | -0.05, 0.08 | 0.68 |
| Fijian of Indian Descent | *ref* | *ref* |  | *ref* | *ref* |  |
| iTaukei | 0.05 | -0.22, 0.32 | 0.70 | 0.00 | -0.26, 0.27 | 0.98 |
| Urban residence | *ref* | *ref* |  | *ref* | *ref* |  |
| Rural residence | 0.09 | -0.11, 0.28 | 0.38 | 0.01 | -0.24, 0.27 | 0.15 |
| Symptoms of URTI | 0.37 | 0.18, 0.55 | <0.01 | 0.28 | 0.09, 0.46 | <0.01 |
| Household cigarette exposure | 0.02 | -0.17, 0.22 | 0.83 |  |  |  |
| Poverty ^c^ | -0.08 | -0.28, 0.11 | 0.41 |  |  |  |
| Participant group |  |  |  |  |  |  |
| Toddlers | *ref* | *ref* |  | *ref* | *ref* |  |
| Young infants | -0.29 | -0.51, -0.06 |  | -0.39 | -0.82, 0.03 |  |
| Young children | 0.65 | 0.44, 0.86 | <0.01 | 0.51 | 0.17, 0.86 | <0.01 |
| Caregivers | 0.37 | -0.01, 0.76 |  | 0.27 | -0.26, 0.79 |  |
| Male | *ref* | *ref* |  |  |  |  |
| Female | -0.05 | -0.23, 0.14 | 0.62 |  |  |  |
| PCV10 vaccinated ^d^ | -0.08 | -0.27, 0.10 | 0.37 | -0.15 | -0.51, 0.20 | 0.40 |
| Antibiotics in past fortnight | 0.40 | -0.12, 0.91 | 0.13 |  |  |  |
| Number of people living in the household | 0.03 | 0.00, 0.05 | 0.03 | 0.01 | -0.02, 0.05 | 0.42 |

URTI: upper respiratory tract infection; ^a^ Only includes participants who were carriers of serotypes not included in PCV10, including non-encapsulated pneumococci; ^b^ Covariates adjusted for physical contact with infants, toddlers, young children, older children, adults; ethnicity, residential location, symptoms of upper respiratory tract infection, participant group, sex, PCV10 vaccination status, and number of people living in the household; ^c^ Family income <FJ$175/wk. [23] ; ^d^ At least two doses of PCV10
